# Impact of text messages in a middle-income country to promote secondary prevention after acute coronary syndrome (IMPACS): A randomized trial

**DOI:** 10.1097/MD.0000000000015681

**Published:** 2019-05-31

**Authors:** Luiz Guilherme Passaglia, Luisa Campos Caldeira Brant, Bruno Ramos Nascimento, Antônio Luiz Pinho Ribeiro

**Affiliations:** a Serviço de Cardiologia e Cirurgia Cardiovascular – Hospital das Clínicas da Universidade Federal de Minas Gerais (UFMG); b Faculdade de Medicina da Universidade Federal de Minas Gerais (UFMG), Belo Horizonte, MG, Brazil.

**Keywords:** acute coronary syndrome, mobile health, risk factors, text messages

## Abstract

**Background:**

Studies that used short message service (SMS) programs as an intervention to promote health care have shown beneficial results in the control of risk factors for ischemic heart disease in patients of high-income countries, but evidence is lacking in low or middle-income countries.

**Aims:**

The purpose of this study is to evaluate whether the use of SMS increases risk factor control within 6 months after discharge by acute coronary syndrome (ACS) in a middle-income country.

**Methods:**

It will be a 2-arm, parallel, double-blind, randomized clinical trial of 160 patients discharged after an ACS from a single center with 6 months of follow-up. The intervention group will receive 4 SMS per week offering advice, motivation and information about medication adherence, increase of regular physical activity, adoption of healthy dietary measures, and smoking cessation (if appropriate). The primary outcome is achieving 4 or 5 points in a risk factor control score, which combines the cluster effect of 5 main modifiable risk factors for ACS [low-density lipoprotein cholesterol, LDL-C <70 mg/dL, blood pressure <140/90 mm Hg, regular exercise (≥5 days/week × 30 minutes of moderate exercise per session), nonsmoker status, and body mass index, BMI <25 kg/m^2^]. Secondary outcomes are plasma LDL-C level, level of physical activity, blood pressure, medication adherence, proportion of nonsmokers, BMI, rehospitalization, cardiovascular death, and death from any cause. This study, as a randomized clinical trial protocol, followed the recommendations of the Standard Protocol Items (SPIRIT).

**Expected outcomes:**

This study aims to provide evidence of whether SMS interventions are effective in improving cardiovascular disease risk factors control in post-ACS patients in a middle-income country.

**ClinicalTrials.gov Identifier:**

NCT03414190 (First posted on January 29, 2018; last update on May 14, 2018) - Retrospectively registered

## Abstract 2

Supplemental Digital Content is available in the text

## 1 Introduction

Cardiovascular disease (CVD) remains the leading cause of death and years of life lost worldwide.^[1]^ In addition, since 1990, there has been a 42% [95% confidence interval (95% CI) 36–48] increase in the absolute number of deaths due to ischemic heart disease globally.^[2]^ In Brazil, CVD has also been the leading cause of mortality since the 1960s and has accounted for a substantial percentage of all hospitalizations. In 2011, CVD was responsible for 31% of all deaths, with ischemic heart disease being the leading cause.^[3]^

Individuals with a prior cardiovascular event have a 5 times greater chance of having another event than people without known CVD.^[4]^ Taking this information into account, a growing body of evidence suggests that adequate management of risk factors substantially reduces unfavorable clinical outcomes, including death, recurrence of ischemic events, and need for revascularization.^[5]^ The World Health Organization estimates that 75% of cardiovascular mortality can be reduced with appropriate changes in lifestyle.^[6]^ When lifestyle interventions are applied to individuals with coronary or other atherosclerotic vascular disease, it is considered secondary prevention.^[7]^

There is a diversified effort to translate cardiovascular science into guidelines to assist health professionals in the management of CVD, for conditions such as acute coronary syndrome (ACS) and its secondary prevention. Despite the increased use of proven effective therapies, adherence to the available recommendations is still below ideal.^[8–10]^ Several telehealth tools, such as text message services—short message service (SMS), can be simple and inexpensive alternatives to encourage healthy life habits and optimize medication adherence.^[11]^

Studies that used SMS as an intervention have shown beneficial results in risk factors control for ischemic heart disease in outpatient settings. Martin et al^[12]^ showed a significant short-term increase in the levels of physical activity of cardiac outpatients. Glynn et al^[13]^ demonstrated similar results on patients seen in rural primary care in an Irish municipality and Wald et al^[14]^ showed an improvement on medication adherence in patients taking blood pressure and lipid-lowering therapies for CVD prevention. Corroborating these findings, using an SMS application, Chow et al^[11]^ found modest improvements in cholesterol levels and moderate reductions on blood pressure, body mass index (BMI), and smoking in patients with coronary artery disease.

Despite the promising results, there is insufficient evidence to draw definite conclusions about the effectiveness of SMS interventions for secondary prevention of CVD, particularly in low- and middle-income countries, where mobile health strategies can have a great impact lowering the costs of health care. Although the access to mobile phone is high in countries such as Brazil, with a density of 112.87 phones/100 inhabitants,^[15]^ the understanding of the messages sent by SMS may not be the same as in high-income countries due to the lower educational stratum of the population.

The IMPACS study will be a 2-arm, parallel, double-blind, and randomized clinical trial. The main purpose of this study is to evaluate whether, in patients who are in secondary prevention of CVD, the use of SMS improves control of CVD risk factors during the 6-month follow-up after discharge by ACS, when compared with the usual treatment. The secondary aim is to develop a wide SMS message bank, semi-personalized, which will include information about lifestyle modifications, medication adherence, and CVD risk factor control. This study, as a randomized clinical trial protocol, followed the recommendations of the Standard Protocol Items (SPIRIT).

## 2 Methods

### 2.1 Participants (study setting and eligibility)

The study population will include patients of the University Hospital of Universidade Federal de Minas Gerais (UFMG's University Hospital), a public and general hospital in southeast Brazil, who were admitted due to diagnosis of ACS. Patients will be also participants of the “Good Practice Program in Cardiology,”^[16]^ a program of the Brazilian Cardiology Society, Ministry of Health (Brazil), and American Heart Association (United States of America).

Inclusion criteria are as follows:

(1) Consecutive patients admitted at the UFMG's University Hospital with primary or secondary diagnosis of ACS and discharged for outpatient follow-up. Confirmed diagnosis of ACS will be defined based on The Third Universal Definition of Myocardial Ischemia.^[17]^
(2) Age ≥18 years, of both sexes;
(3) Patients who are able to receive SMS in their own mobile phone.

Exclusion criteria are as follows:

(1) Refusal or inability to sign the informed consent;
(2) Complete illiteracy.

### 2.2 Interventions

The usual care group (IMPACS control group) will receive standard discharge treatment and standard hospital follow-up. They will monthly receive text messages thanking for their participation in the trial and reminders of trial appointment.

The intervention group (IMPACS intervention group) will receive the usual post-discharge care for ACS, instructions and information, as well as the SMS intervention program. The SMS program will include a variety of topics, such as standard follow-up care reminders and general self-management and healthy habits texts to inform and engage patients in care. The Intervention will run for 6 months and the program will consist of 4 modules, according to baseline characteristics of participants:

(1) Module 1: nonsmokers and free of diabetes;
(2) Module 2: nonsmokers and diabetic patients;
(3) Module 3: smokers and nondiabetic patients;
(4) Module 4: smokers and diabetic patients.

Modules 2 and 4 are subdivided into 2 according to whether or not diabetic patients are in use of insulin. Texts will be sent out 4 times per week for 180 days at pre-established times, with the first SMS being sent immediately after hospital discharge. All participants included in the same module will receive the same texts and in the same order, regardless of the allocation time in the study. No cross-over between groups is expected.

All participants will also be followed by the UFMG's University Hospital Coronary Artery Disease outpatient clinic and rehabilitation program, unless they choose otherwise. IMPACS researchers will be independent of attending physicians and will not interfere with patient care.

#### 2.2.1 Technological development

Dedicated software was developed by The Telehealth Center of UFMG's University Hospital to send 1-way SMS between server (Windows) and participant's mobile phone.^[18]^ The software has a bank of text messages that allows identification and scheduling for submission of SMS on predetermined dates. To test the software developed and the initial acceptability of text messages sent, a pilot study was conducted and it is described in Appendix 1.

#### 2.2.2 Text messages

The messages that will be used in this study were developed by the research group, offering advice, motivation and information about medication adherence, increase of regular physical activity, adoption of healthy dietary habits, and smoking cessation (if appropriate). The content of the messages is based on the Brazilian Society of Cardiology Guidelines, available online.^[19]^ Messages will be semi-personalized because the objective is to combine general information with personalized content, using information provided in baseline questionnaires. The bank of text messages, after being developed, was reviewed by individuals not involved in the study to check for language issues and evaluation of understanding. The main goal was to make messages easy to read and understand, for all cultural and social levels. Text messages content according to each module is summarized in Table 1 and examples of text messages sent to the IMPACS intervention group can be seen in Table 2.

**Table 1:**
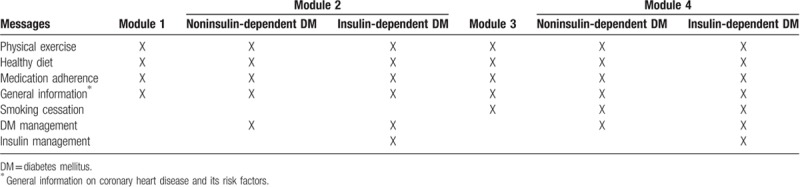
Text messages according to modules based on characteristics of participants.

**Table 2:**
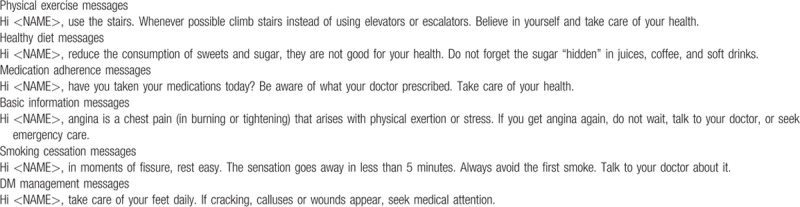
Examples of text messages sent to the IMPACS intervention group.

At study entry, all participants will be given brief training (three to five minutes) of how to read text messages, save, and delete them, if necessary. The Telehealth Center of UFMG's University Hospital will manage the SMS through a computerized messaging engine, and because of that, participants will be instructed not to respond to them. Interactive communication will only occur via e-mail, if the patient shows an intention to discontinue his/her participation in the study. Messages will be sent at no cost to the participants.

### 2.3 Outcomes

Participants will have outcomes measured at 6 months (±1 month) after hospital discharge, in the follow-up appointment pre-scheduled by the study's researchers. Researchers blinded to treatment allocation will collect the data.

(1) Primary endpoint: The primary outcome is achieving 4 or 5 points in a risk factor control score, which combine the cluster effect of 5 main modifiable risk factors for ACS [low-density lipoprotein cholesterol - LDL-C <70 mg/dL, blood pressure <140/90 mm Hg, regular exercise (≥5 days/week × 30 minutes of moderate exercise per session), nonsmoker status, and BMI <25 kg/m^2^].
(2) Secondary endpoints: Plasma LDL-C levels, objective level of physical activity, blood pressure, medication adherence, proportion of nonsmokers, BMI, death from any cause, rehospitalization, and cardiovascular death.

### 2.4 Sample size

A sample size of 141 patients was estimated, increasing to 160 to allow for a 15% loss to follow-up, 2-tailed and at a 5% significance level, would have 80% power to detect a difference of at least 19% between the intervention and the control groups in achieving 4 or more of the 5 modifiable risk factors listed above (Risk Factor Control Score), based on the findings of the study by Chow et al.^[11]^ An interim analysis before the end of the patient allocation will be done to evaluate the follow-up losses with the objective of reassessing the sample size initially considered.

### 2.5 Allocation and blinding

After obtaining written informed consent, the data of each patient will be entered into an online database. To reduce predictability of a random sequence, a blocking randomization will be provided in blocks of 4 patients following the date of patient enrollment. The computerized randomization program is only accessible to administrators of the Telehealth Center of the UFMG's University Hospital. The random allocation sequence will follow a uniform 1:1 ratio and the researchers, data collectors, and attending physicians will be blind to the treatment allocation (double blinding).

### 2.6 Data collection methods

The eligible patients will be identified by daily assessment in the Coronary Intensive Care Unit of UFMG's University Hospital and followed up throughout the hospitalization for data collection. All data collectors will undergo specific training for understanding and systematically applying the study protocol.

Demographic, educational, socioeconomic, and clinical characteristics of the study participants will be collected at baseline. At this stage, ACS type and severity criteria, diagnostic and therapeutic procedures performed in the in-hospital phase, and prescribed medications at hospital discharge will also be collected. Before hospital discharge (up to 48 hours before discharge), patient weight, height, heart rate, and blood pressure will be additionally measured for future analysis.

The participant timeline is described in Appendix 02 and the flow chart of study design in Table 3.

**Table 3:**
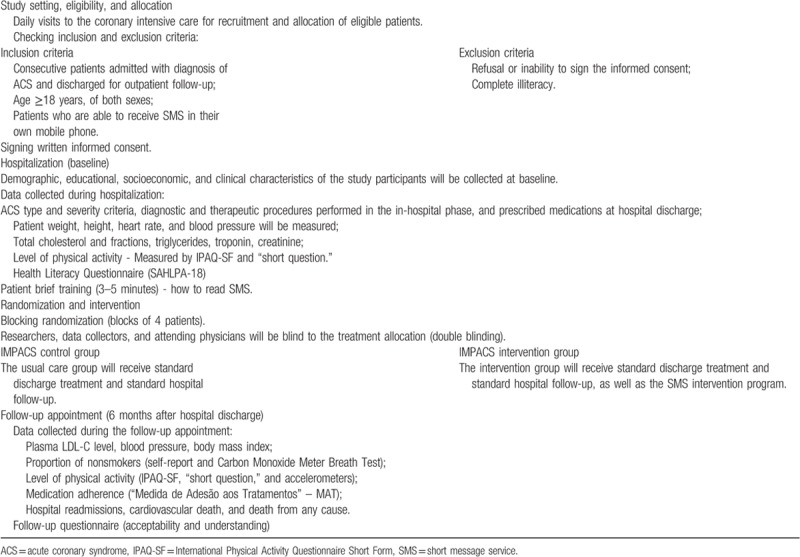
Flow chart of study design.

The data collection protocols are briefly summarized in the following:

(1) Plasma LDL-C level: measured at baseline and 6 months after hospital discharge (before the outpatient appointment), after 12 hours of fasting, in the same laboratory. Measurement of total cholesterol, triglycerides, and high-density lipoprotein cholesterol will be done with CHOL VITROS Chemistry Products slides from Ortho Clinical Diagnostics (VITROS 5.1 and VITROS 5.600/colorimetric method). For LDL-C dosage, the value is generally calculated. When triglycerides value >400 mg/dL, we will perform direct dosing of LDL-C using the reagent LDL VITROS Chemistry Products from Ortho Clinical Diagnostics (VITROS 5.1 and VITROS 5.600 / End point Method).
(2) Level of physical activity: measured by the International Physical Activity Questionnaire Short Form (IPAQ-SF) ^[20]^ at baseline and 6 months after hospital discharge. The measure will be validated in one-fifth of the participants by using accelerometers (Actigraph wGT3X-BT; Pensacola, FL). A question about whether the patient is performing scheduled physical activity following the recommendation of the Brazilian Society of Cardiology will also be included in the follow-up questionnaire.^[21]^
(3) Blood pressure: measured at baseline and 6 months after hospital discharge by the same automatic blood pressure device (OMRON model HEM-705 CP Intellisense) and OMRON blood pressure cuffs up to 48 hours before hospital discharge and in the follow-up visits. Three resting measurements will be made, with the patient in the seated position, with the arm supported. The mean of the last 2 readings will be considered for data analysis.
(4) Medication adherence: measured at 6 months after hospital discharge via “Medida de Adesão aos Tratamentos” – MAT,^[22]^ a validated instrument composed of 6 items that evaluate the behavior of the individual in relation to daily use of medicines. The answers are obtained by means of a 6-point ordinal scale that varies from “always” [1] to “never” [6]. The values obtained with the answers to the 6 items are summed and divided by the number of items (values vary from 1 to 6). Subsequently, values 5 and 6 are computed as 1 (adherent) and the others are computed as 0 (nonadherent).
(5) Proportion of nonsmokers: measured at 6 months after hospital discharge by self-report. The participant will answer the following question: “Are you smoking after hospitalization?”, for which he/she could answer “Yes” or “No.” The answer will be also confirmed by a Carbon Monoxide Meter Breath Test (piCO Smokelyzer, Kent, UK). This is an indirect and noninvasive measure of blood carboxyhemoglobin. The device directly measures carbon monoxide (ppm) and carboxyhemoglobin is a calculation based on clinical evidence. The values obtained by the device are 0 to 6 (nonsmoker range), 7 to 19 (light smoking range), and 20 or more (heavy smoking range).^[23,24]^
(6) BMI: measured at baseline and 6 months after hospital discharge. BMI will be calculated by weight measured in kilograms divided by height in square meters.
(7) Rehospitalization: measured at 6 months by means of self-report and medical discharge records.
(8) Cardiovascular death and death from any cause: measured at 6 months by report of relatives and confirmed by death certificate or medical records.
(9) Health literacy questionnaire: measured at baseline. A version consisting of 18 items of The Short Assessment of Health Literacy for Portuguese speaking Adults (SAHLPA-18) ^[25]^ will be applied to participants at the baseline.
(10) Follow-up questionnaire: a follow-up questionnaire with self-report of reading messages will be applied as well as questions about acceptability and understanding (Appendix 3).

### 2.7 Data management and monitoring

Data management will be done by the Telehealth Center of UFMG's University Hospital. Data entry will be made through a password-protected, web-based interface by a registered team and double-checking for verification of data consistency will be performed. The study's steering committee (LGP, LCCB, ALPR) has the overall responsibility for the conduction and periodic monitoring of the study.

### 2.8 Statistical methods

Analysis will be performed according to the intention-to-treat principle. For the baseline characteristics, continuous variables will be summarized as mean ± SD or as median and first and third quartiles (Q1, Q3), and groups compared using Student *t* tests or Mann–Whitney test, based on the distribution pattern. Categorical variables will be expressed as proportions and 95% CIs and groups compared by Chi-square test. The primary outcome will be compared between groups using Chi-square test. We will also analyze effects across subgroups, such as sex, age, or other covariates of interest, using logistic regression models. The criterion for statistical significance will be set at α = 0.05 and SPSS Statistics for Windows (Version 20.1; IBM Corp., Armonk, NY) will be used for the analysis.

### 2.9 Ethics and dissemination

Patients will be asked if they wish to participate in the study while hospitalized. After screening for inclusion and exclusion criteria, eligible patients will receive written an oral information about the study. Written and informed consent will be obtained from all participants before allocation. The ethical approval for this study has been obtained from The Medical Ethics Committee of the Universidade Federal de Minas Gerais (Number: 2,054,294 dated August 03, 2017). Any changes in the protocol during the trial that may affect the conduct of the trial, safety, and the benefit to patients will require a formal amendment to the initial protocol and be immediately communicated to The Medical Ethics Committee. The findings of this study will be published and disseminated via scientific forums, with no restrictions.

## 3 Discussion and conclusion

The “IMPACS” study is an innovative study, as it evaluates the implementation of an effective and simple strategy in the secondary prevention of CVD in a middle-income country. Two important issues make this study different from others who used SMS as a telehealth tool: the range of approaches and the measurement of health literacy.

The messages address several essential conditions for CVD prevention at the same time: medication adherence, regular physical exercise, healthy dietary habits, smoking cessation (if appropriate), diabetes management (if appropriate), and general information on coronary heart disease, all of them with a language adapted to cultural and social level of the target population. In order to compare the results of the IMPACS study with those found in previous studies of SMS intervention in high-income countries, the assessment of health literacy is a crucial factor.

In conclusion, the IMPACS study aims to provide information, by randomized controlled data, whether SMS interventions is effective in increasing CVD risk factors control in patients post-ACS in a middle-income country.

## Acknowledgments

The authors acknowledge Verizon for providing the carbon monoxide meter and accelerometers through the PROVAR (Programa de Rastreamento da VAlvopatia Reumática) program. We would like to thanks Giovana Zoboli Semabukuro, Giovanna Ribas Passagli, Gustavo Couto Pereira da Silva, Lucas Nevez Vaz, Lorhayne Kerley Capuchinho Scalioni, Mariana Martins Pires, and Mariana Figueiredo Simões by the direct contribution in the data collection of the IMPACS study.

## Author contributions

LGP, ALPR, and LCCB have participated in study design. LGP is responsible by the collection and management of data. All of them will be responsible by analysis and interpretation of data. All of them have seen and approved the submitted manuscript, which reports unpublished work not under consideration elsewhere. LGP and LCCB have done the literature review, article selection, and designed the scope of the article. All authors contributed in the writing of the manuscript. ALPR and BRN reviewed the final version. All authors have read and approved the manuscript.

**Conceptualization:** Luiz Guilherme Passaglia, Luisa Campos Caldeira Brant, Antônio Luiz Pinho Ribeiro.

**Data curation:** Luiz Guilherme Passaglia.

**Formal analysis:** Luiz Guilherme Passaglia, Antônio Luiz Pinho Ribeiro.

**Funding acquisition:** Antônio Luiz Pinho Ribeiro, Bruno Ramos Nascimento.

**Investigation:** Luiz Guilherme Passaglia.

**Methodology:** Luiz Guilherme Passaglia, Luisa Campos Caldeira Brant.

**Project administration:** Luiz Guilherme Passaglia.

**Supervision:** Luiz Guilherme Passaglia, Luisa Campos Caldeira Brant, Antônio Luiz Pinho Ribeiro, Bruno Ramos Nascimento.

**Writing – original draft:** Luiz Guilherme Passaglia.

**Writing – review & editing:** Luiz Guilherme Passaglia, Luisa Campos Caldeira Brant, Antônio Luiz Pinho Ribeiro, Bruno Ramos Nascimento.

## Supplementary Material

Supplemental Digital Content

## References

[1] GBD 2016 Causes of Death Collaborators. Global, regional, and national age-sex specific mortality for 264 causes of death, 1980-2016: a systematic analysis for the Global Burden of Disease Study 2016. Lancet 2017;390:1151–210. 28919116 10.1016/S0140-6736(17)32152-9 PMC5605883

[2] Shepard D VanderZanden A Moran A Ischemic heart disease worldwide, 1990 to 2013: estimates from the Global Burden of Disease Study 2013. Circ Cardiovasc Qual Outcomes 2015;8:455–6. 26152681 10.1161/CIRCOUTCOMES.115.002007 PMC4589220

[3] Ribeiro AL Duncan BB Brant LC Cardiovascular health in Brazil: trends and perspectives. Circulation 2016;133:422–33. 26811272 10.1161/CIRCULATIONAHA.114.008727

[4] Kerr AJ Broad J Wells S Should the first priority in cardiovascular risk management be those with prior cardiovascular disease? Heart 2009;95:125–9. 18381374 10.1136/hrt.2007.140905

[5] Smith SC Jr Benjamin EJ Bonow RO AHA/ACCF secondary prevention and risk reduction therapy for patients with coronary and other atherosclerotic vascular disease: 2011 update: a guideline from the American Heart Association and American College of Cardiology Foundation endorsed by the World Heart Federation and the Preventive Cardiovascular Nurses Association. J Am Coll Cardiol 2011;58:2432–46. 22055990 10.1016/j.jacc.2011.10.824

[6] 65th World Health Assembly closes with new global health measures. Cent Eur J Public Health 2012; 20:163–164. 22966746

[7] Smith SC Jr Benjamin EJ Bonow RO AHA/ACCF secondary prevention and risk reduction therapy for patients with coronary and other atherosclerotic vascular disease: 2011 update: a guideline from the American Heart Association and American College of Cardiology Foundation. Circulation 2011;124:2458–73. 22052934 10.1161/CIR.0b013e318235eb4d

[8] Yan RT Yan AT Tan M Underuse of evidence-based treatment partly explains the worse clinical outcome in diabetic patients with acute coronary syndromes. Am Heart J 2006;152:676–83. 16996832 10.1016/j.ahj.2006.04.002

[9] Kotseva K Wood D De Backer G EUROASPIRE III. Management of cardiovascular risk factors in asymptomatic high-risk patients in general practice: cross-sectional survey in 12 European countries. Eur J Cardiovasc Prev Rehabil 2010;17:530–40. 20577089 10.1097/HJR.0b013e3283383f30

[10] Chowdhury R Khan H Heydon E Adherence to cardiovascular therapy: a meta-analysis of prevalence and clinical consequences. Eur Heart J 2013;34:2940–8. 23907142 10.1093/eurheartj/eht295

[11] Chow CK Redfern J Hillis GS Effect of lifestyle-focused text messaging on risk factor modification in patients with coronary heart disease: a randomized clinical trial. JAMA 2015;314:1255–63. 26393848 10.1001/jama.2015.10945

[12] Martin SS Feldman DI Blumenthal RS mActive: a randomized clinical trial of an automated mhealth intervention for physical activity promotion. J Am Heart Assoc 2015;4:pii: e002239. 10.1161/JAHA.115.002239 PMC4845232 26553211

[13] Glynn LG Hayes PS Casey M Effectiveness of a smartphone application to promote physical activity in primary care: the SMART MOVE randomised controlled trial. Br J Gen Pract 2014;64:e384–91. 24982490 10.3399/bjgp14X680461 PMC4073723

[14] Wald DS Bestwick JP Raiman L Randomised trial of text messaging on adherence to cardiovascular preventive treatment (INTERACT trial). PLoS One 2014;9:e114268. 25479285 10.1371/journal.pone.0114268 PMC4257733

[15] Anatel. Agência Nacional de Telecomunicações - Anatel. Available at: http://www.anatel.gov.br/institucional/ Accessed June 1, 2018.

[16] Cardiologia SBd. Programa Boas Práticas Clínicas em, Cardiologia, Sociedade Brasileira de Cardiologia. 2015 Available at: http://www.cardiol.br/boaspraticasclinicas Accessed June 1, 2018.

[17] Thygesen K Alpert JS Jaffe AS Third universal definition of myocardial infarction. Eur Heart J 2012;33:2551–67. 22922414 10.1093/eurheartj/ehs184

[18] Gusmão LL Ribeiro AL Souza-Silva MVR Implementation of a text message intervention to promote behavioural change and weight loss among overweight and obese Brazilian primary care patients. J Telemed Telecare 2018;[Epub ahead of print]. 10.1177/1357633X18782092 29950150

[19] Cardiology BSo. Scientific Publications of the Brazilian Society of Cardiology Published From 2014 to 2019. Accessed August 1, 2017.

[20] Lee PH Macfarlane DJ Lam TH Validity of the International Physical Activity Questionnaire Short Form (IPAQ-SF): a systematic review. Int J Behav Nutr Phys Act 2011;8:115. 22018588 10.1186/1479-5868-8-115 PMC3214824

[21] Simao AF Precoma DB Andrade JP [I Brazilian Guidelines for cardiovascular prevention]. Arq Bras Cardiol 2013;101 6 suppl 2:1–63. 10.5935/abc.2013S012 24554026

[22] Delgado ABLM Contribution to the concurrent validation of a treatment compliance measure [Portuguese]. Psychology 2001;1:81–100.

[23] Jarvis MJ Belcher M Vesey C Low cost carbon monoxide monitors in smoking assessment. Thorax 1986;41:886–7. 3824275 10.1136/thx.41.11.886 PMC460516

[24] Christenhusz L de Jongh F van der Valk P Comparison of three carbon monoxide monitors for determination of smoking status in smokers and nonsmokers with and without COPD. J Aerosol Med 2007;20:475–83. 18158719 10.1089/jam.2007.0606

[25] Apolinario D Braga Rde C Magaldi RM Short assessment of health literacy for Portuguese-speaking adults. Rev Saude Publica 2012;46:702–11. 22782124 10.1590/s0034-89102012005000047

