# Supplementary material for: Impact of text messages in a middle-income country to promote secondary prevention after acute coronary syndrome (IMPACS): A randomized trial
Source: Medicine (Baltimore). 2019 May 31;98(22):e15681. doi: 10.1097/MD.0000000000015681 (PMC6709304; doi:10.1097/MD.0000000000015681)
Supplement: Supplemental Digital Content [file medi-98-e15681-s001.docx]

**APPENDIX 01 – PILOT STUDY**

In order to test the software developed for the study and the initial acceptability of text messages sent, a pilot study was conducted between October and November 2017. Ten patients hospitalized for Acute Coronary Syndrome in the UFMG’s University Hospital, who signed a specific written informed consent, received 4 to 7 messages per day for 3 consecutive days after hospital discharge. By telephone, a questionnaire on the usefulness and acceptability of text messages was applied with the following questions:

1. How many text messages per day did you receive?
2. How many messages per day did you read?
3. Was I able to easily access the messages on my mobile phone?
4. Did I understand what the messages say?
5. Will the messages help change my daily habits (diet, exercise, etc.)?
6. Were the messages definitely useful for my treatment?

The first two questions were answered by “message numbers” and questions from three to six were answered according to the following classification: I totally agree, or I agree more than disagree, or I disagree more than I agree, or I strongly disagree.

As result, of the 10 patients included: one patient did not answer the calls; one patient answered he did not received any of the messages (although the system confirmed the sending); one patient had difficulty to answer the questions by phone call (but confirmed to be read the messages); and 7 patients answered that they received all the messages sent, read all of them and fully agreed with the questions from three to six of the questionnaire. Based on these results, we concluded: (1) the software developed was capable to send the scheduled SMS; and (2) for the sample size calculation, a loss of approximately 15% of the sample could occur in the follow-up.

**APPENDIX 02 – PARTICIPANT TIMELINE**

| **Study Period** | **Dates** | | | |
| --- | --- | --- | --- | --- |
|  | **Enrollment (November 2017 to January 2019)** | **Allocation (November 2017 to January 2019)** | **Intervention (November 2017 to August 2019)** | **Follow-up visit (May 2018 to September 2019)** |
| **Enrollment** |  | | | |
| Eligility | X |  |  |  |
| Informed Consent | X |  |  |  |
| Allocation |  | X |  |  |
| Intra-hospital Data Collection |  | X |  |  |
| **Interventions** |  | | | |
| Sending Text Messages |  |  | X |  |
| **Assessments** |  | | | |
| Follow-up visit (measurement of outcomes) |  |  |  | X |

**APPENDIX 03 –** Usefulness and Acceptability Questionnaire for Text Messages

**Questions**

1) Did I receive text messages from IMPACS study on my cell phone?

2) Have I read the messages I received from the IMPACS Study on my cell phone?

3) Was I able to easily access the messages on my cell phone?

4) Did I understand the information that the text messages is providing?

5) Will the messages help me change my daily habits (like diet or exercise routine)?

6) Were the messages definitely useful for my heart treatment?

The response options were “Yes, No” for item 1.

The response options were “strongly agree, agree more than disagree, disagree more than agree, totally disagree” for items 2, 3, 4, 5, and 6.
